# Nicotine Induces Resistance to Erlotinib Therapy in Non-Small-Cell Lung Cancer Cells Treated with Serum from Human Patients

**DOI:** 10.3390/cancers11030282

**Published:** 2019-02-27

**Authors:** Tatsuya Imabayashi, Junji Uchino, Hisayuki Osoreda, Keiko Tanimura, Yusuke Chihara, Nobuyo Tamiya, Yoshiko Kaneko, Tadaaki Yamada, Koichi Takayama

**Affiliations:** 1 Department of Pulmonary Medicine, Kyoto Prefectural University of Medicine Graduate School of Medical Science, Kyoto 602-0841, Japan; imabayas@koto.kpu-m.ac.jp (T.I.); keiko-t@koto.kpu-m.ac.jp (K.T.); c1981311@koto.kpu-m.ac.jp (Y.C.); koma@koto.kpu-m.ac.jp (N.T.); kaneko-y@koto.kpu-m.ac.jp (Y.K.); tayamada@koto.kpu-m.ac.jp (T.Y.); takayama@koto.kpu-m.ac.jp (K.T.); 2 Department of Internal Medicine, Self Defense Forces Fukuoka Hospital, Fukuoka 816-0826, Japan; osore@isis.ocn.ne.jp

**Keywords:** cotinine, nicotine, non-small-cell lung cancer (NSCLC), EGFR, erlotinib resistance

## Abstract

Previously, we reported that nicotine reduces erlotinib sensitivity in a xenograft model of PC9, an epidermal growth factor receptor-tyrosine kinase inhibitor (EGFR-TKI)-sensitive non-small-cell lung cancer cell line. The present study examined whether smoking induces erlotinib resistance in vitro. We assessed resistance to EGFR-TKIs by treating cancer cell lines with erlotinib, afatinib, or osimertinib, and serum collected from smokers within 30 min of smoking and that from a non-smoker as a control. We also assessed erlotinib resistance by treating PC9 cells exposed to serum from a smoker or a non-smoker, or serum from an erlotinib user. Treatment of the cancer cell lines with serum from smokers induced significant erlotinib resistance, compared with the control (*p* < 0.05). Furthermore, serum samples with a high concentration of cotinine (a nicotine exposure indicator) demonstrated stronger erlotinib resistance than those with low concentrations. Similar to the observations with erlotinib treatment of cell lines, the analysis of serum from erlotinib users revealed that smokers demonstrated significantly reduced sensitivity to erlotinib (*p* < 0.001). In conclusion, our present results support the hypothesis that smoking contributes to resistance to erlotinib therapy in non-small-cell lung cancer.

## 1. Introduction

Smoking is a major risk factor for lung cancer, 85% of which is non-small-cell lung cancer (NSCLC) [1]. One of the primary components of tobacco is nicotine, which is known to promote cancer cell growth, metastasis, and resistance to chemotherapy. Nicotine is believed to exert these effects by binding to nicotinic acetylcholine receptors (nAchRs) expressed on lung cancer cells, thereby activating signaling pathways such as the Phosphoinositide 3-kinase/ Protein Kinase B (PI3K/AKT), Extracellular Signal-regulated Kinase (ERK) 1/2, Mitogen-activated Protein Kinase (MAPK), Mitogen-activated protein kinase kinase (MEK), nuclear factor-kappa B (NFκB), β-arrestin-1, Src kinase, and Rb-Raf-1 signaling pathways, and consequently triggering cell survival and proliferation, angiogenesis, invasion, epithelial-mesenchymal transition, and inhibition of apoptosis [2,3,4,5,6,7,8,9,10,11,12,13,14,15,16,17]. While there are various subunits (α [1–10], β [1–4], δ, γ, and ε) of nAchRs [2], we have focused on the α1 subunit to investigate the association between nicotine and lung cancer.

We previously reported that α1nAchR is expressed in the epidermal growth factor receptor (EGFR) mutation-positive NSCLC cell lines (PC9 and HCC827) [18,19]. Furthermore, we demonstrated that the action of nicotine on α1nAchR in murine xenograft models of both cell lines activates EGFR signaling pathways via PI3K/AKT and ERK1/2, and reduces sensitivity to erlotinib, a typical EGFR-tyrosine kinase inhibitor (TKI) [18,19]. The present study aimed to examine whether smoking induces resistance to erlotinib, using human serum obtained from smokers and non-smokers.

## 2. Results

### 2.1. Serum Cotinine Levels of All Subjects

Table 1 shows the age, sex, smoking history, and serum cotinine levels of the four smokers and one non-smoker. Heavy smokers showed higher cotinine levels than light smokers. Smoker No. 4 showed the highest cotinine level at 488.4 ng/mL.

### 2.2. Treatment of PC9 and HCC827 Cells with Serum from the Reduced Sensitivity of Smokers to Erlotinib

**Figure 1:**
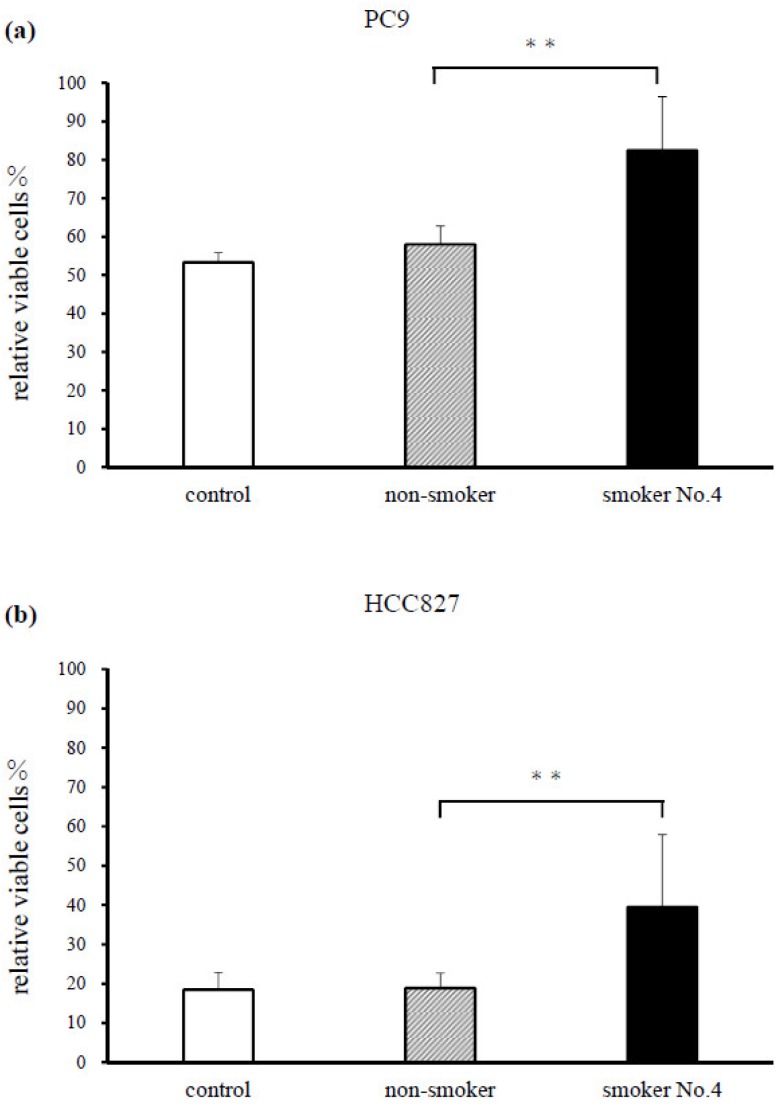
Treatment of (**a**) PC9 and (**b**) HCC827 cells with serum from a smoker reduces sensitivity to erlotinib therapy. Treatment of cells for 72 h with 1 μM erlotinib and serum from smoker No. 4 (serum cotinine level: 488.4 ng/mL) resulted in a significant reduction of sensitivity to erlotinib compared with serum from a non-smoker control (serum cotinine level: 0.6 ng/mL) in both cell lines (** *p* < 0.001). Cell survival was assessed by using a cell-counting kit (CCK)-F. Results are means ± SEM of four independent experiments.

The erlotinib-treated (1 μM) PC9 and HCC827 cell lines demonstrated significantly reduced erlotinib sensitivity when treated with serum from smoker No. 4, compared with that from the non-smoker (*p* < 0.001, Figure 1a,b).

**Figure 2:**
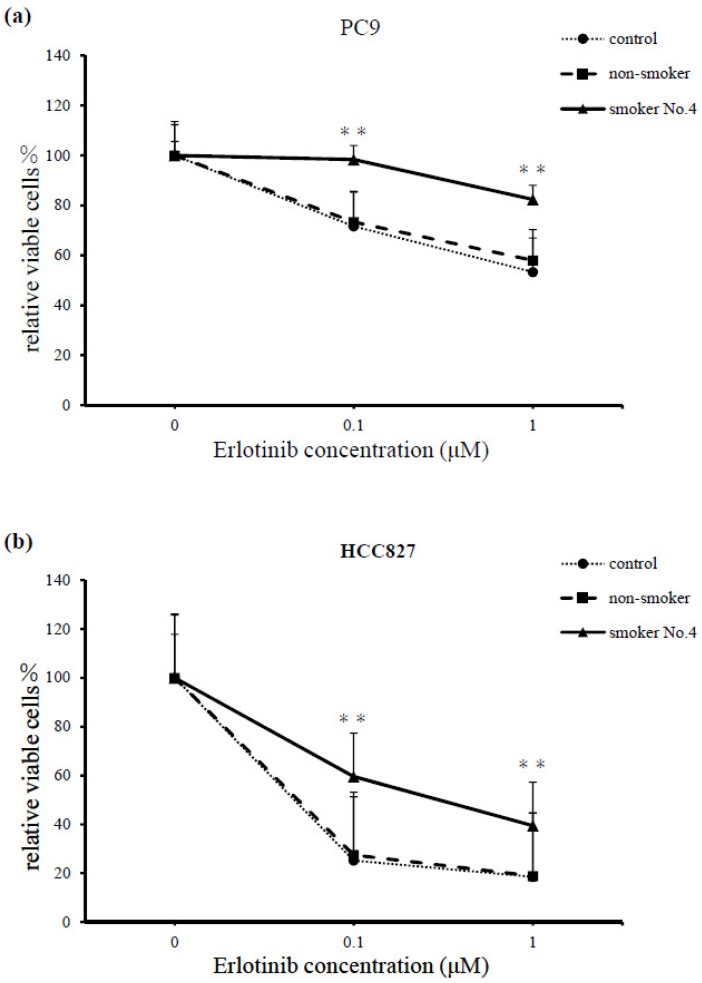

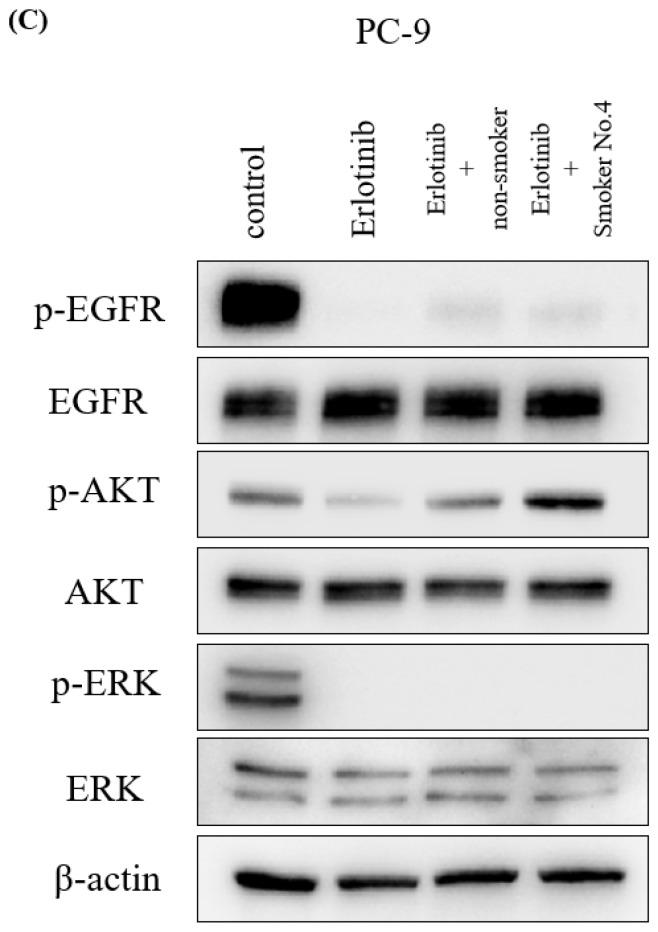
Comparisons of (**a**) PC9 and (**b**) HCC827 cell lines cultured for 72 h with various concentrations of erlotinib (0, 0.1, and 1 μM), and serum from the non-smoker and smoker No. 4. Serum from the smokers demonstrated significant resistance to erlotinib treatment at all concentrations in both cell lines, compared with serum from the non-smoker (at 1 μM erlotinib in the PC9 cell, *p* = 0.0018; for all other comparisons, *p* < 0.001). Cell survival was assessed using a cell counting kit (CCK)-F. Results are means ± SEM of four independent experiments. (**c**) Immunoblot analysis of PC9 cells incubated with erlotinib (1 μM), and serum from the non-smoker or smoker No. 4 for 1 h. The combination of erlotinib with serum from the smoker elevated the protein levels of the phosphorylated AKT (Ser 473) considerably. AKT phosphorylation was inhibited by erlotinib and serum from the non-smoker. Erlotinib inhibited the phosphorylation of EGFR and ERK, independent of serum addition. The control is untreated cells.

At various concentrations of erlotinib (0; 0.1; and 1 μM), serum from smoker No. 4 reduced the cell-killing effect of erlotinib in both PC9 and HCC827 cell lines, compared with the serum from the non-smoker (at erlotinib 1 μM in PC9 cells, *p* = 0.0018; for all other comparisons, *p* < 0.001, Figure 2a,b).

To identify the signaling mechanisms of smoking-induced resistance to erlotinib, we then assessed the protein levels of PC9 cells cultured with erlotinib (1 μM) and serum from the non-smoker or smoker No. 4 for 1 h. The combination of erlotinib and serum from smoker No. 4 elevated the protein levels of phosphorylated AKT (Ser 473) considerably, while AKT phosphorylation was inhibited in cells treated with erlotinib and serum from the non-smoker. Erlotinib inhibited the phosphorylation of EGFR and ERK, independent of serum addition (Figure 2c).

**Figure 3:**
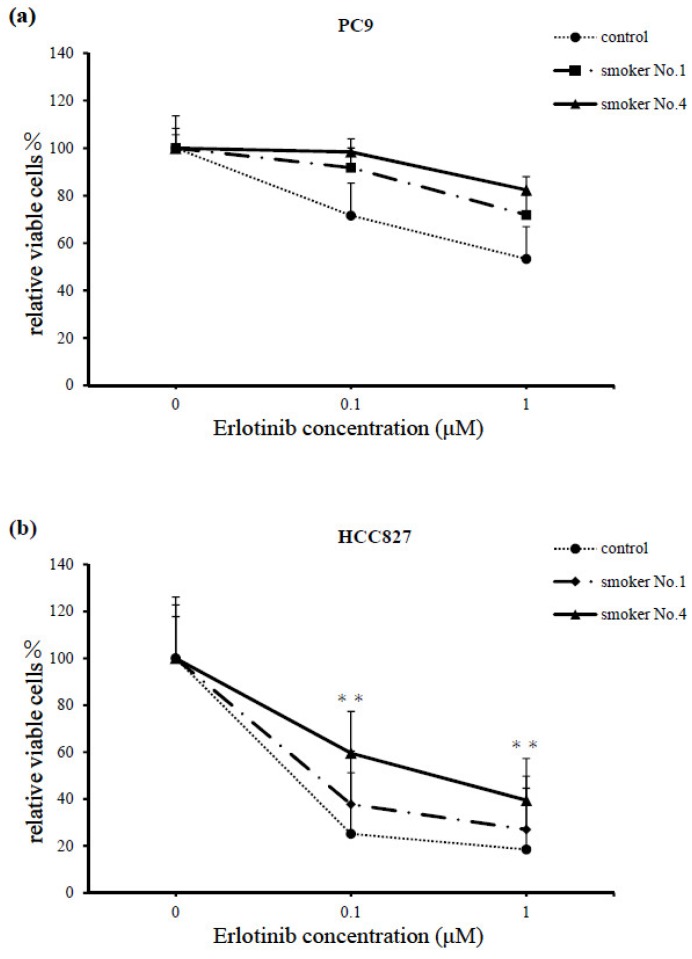
Comparison between smokers No. 1 and 4 with the lowest and highest serum cotinine levels (33.0 and 488.4 ng/mL), respectively. Serum with the highest levels showed stronger resistance to erlotinib therapy over 72 h. (**a**) PC9 cells treated with 0.1 and 1 μM erlotinib, *p* = 0.8077 and 0.4242, respectively. (**b**) HCC827 cells treated with 0.1 and 1 μM erlotinib, ** *p* < 0.001. Cell survival was assessed using a cell counting kit (CCK)-F. The Results are means ± SEM of four independent experiments.

Additionally, the smoker with the highest serum cotinine level (No. 4) showed greater resistance to erlotinib treatment than the smoker with the lowest serum cotinine level (No. 1, 33.0 ng/mL). Specifically, the resistance was greater in HCC827 cells at erlotinib concentrations of 0.1 and 1 μM (*p* < 0.001), and in PC9 cells at erlotinib concentrations of 0.1 and 1 μM (*p* = 0.8077 and 0.4242, respectively; Figure 3a,b). In this experiment, we think that the difference in cell survival between PC-9 and HCC 827 was due to differential dependence on the EGFR signal in the cells lines. However, it is worth noticing that although the difference was not significant, the PC-9 cell line also showed a tendency for increased survival when treated with the serum of patient No. 4. We therefore think that nicotine ingestion influences the therapeutic effects of erlotinib in both cell lines.

### 2.3. Treatment of PC9 Cells with Serum from Smokers Reduced Sensitivity to Afatinib and Osimertinib

**Figure 4:**
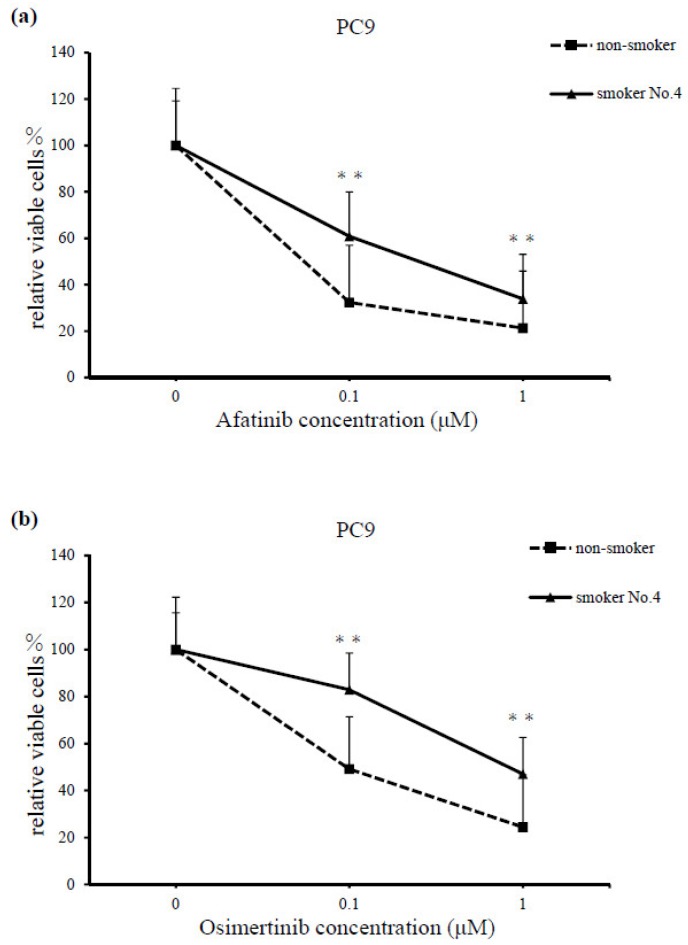
Comparisons of PC9 cell lines cultured for 72 h with various concentrations of (**a**) afatinib and (**b**) osimertinib (0, 0.1, and 1 μM) and serum from the non-smoker and smoker No. 4. Serum from the smoker demonstrated significant resistance to afatinib and osimertinib treatment, compared with serum from non-smoker (*p* < 0.001). Cell survival was assessed using a cell counting kit (CCK)-F. Results are means ± SEM of five independent experiments.

At various concentrations of afatinib and osimertinib (0, 0.1, and 1 μM), the serum from smoker No. 4 reduced the cell-killing effects of both drugs in the PC9 cell line, compared with the serum from the non-smoker (*p* < 0.001; Figure 4a,b).

### 2.4. PC9 Cells Treated with Serum from a Smoker Showed Erlotinib Resistance When Further Treated with Serum from the Erlotinib User

**Figure 5:**
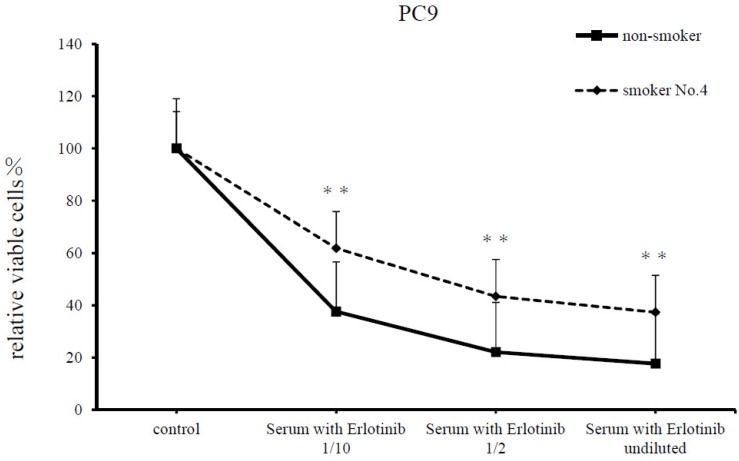
Treatment of PC9 cells with serum from smoker-induced erlotinib resistance following treatment with serum from an erlotinib user. Analysis of PC9 cells treated with serum from a non-smoker or smoker No. 4 for 24 h, followed by serum from erlotinib users (1:10 or 1:2 dilutions, or undiluted) for 120 h. Serum from smokers significantly reduced erlotinib sensitivity (** *p* < 0.001). Cell survival was assessed using a cell counting kit (CCK)-F. Results are means ± SEM of four independent experiments.

PC9 cells cultured with serum from a patient treated with erlotinib showed reduced cell numbers compared with untreated cells. However, when additionally treated with serum from smoker No. 4, the cell inhibitory effect was significantly reduced compared with additional treatment with serum from the non-smoker (*p* < 0.001, Figure 5).

## 3. Discussion

For patients with NSCLC, continued smoking, smoking cessation failure, and inhalation of secondhand smoke may exacerbate the risks of tumor progression, resistance to therapy, post-therapy recurrence, and death [20,21,22,23]. Although molecular targeted therapy with EGFR-TKIs such as erlotinib has dramatically improved the outcomes of patients with EGFR mutation-positive NSCLC, smoking is known to negatively impact the effects of EGFR-TKI therapy. Smokers show poor survival rates in EGFR-TKI therapy compared to those who have never previously smoked [24,25,26,27]. This is likely because smokers display rapid erlotinib clearance that is 24% faster than that in non-smokers, and they use 300 mg to obtain the same area under the curve values compared with the normal dose of 150 mg used in non-smokers. Furthermore, the action of nicotine activates EGFR signaling pathways via α1nAchR, thereby inducing resistance to erlotinib therapy [28].

Although nicotine exhibits age- and race-related differences in its effects [29,30,31], it is seldom measured as a quantitative indicator of smoking, because it is rapidly metabolized by CYP2A6 [32], with a half-life of several hours [33]. A smoker who weighs 68 kg and who smokes 20 cigarettes per day is considered to have a serum nicotine level of 1 μM [14]. Because blood nicotine levels peak at the end of smoking a cigarette and decline rapidly over the next 30 min due to tissue distribution [34], blood was sampled from smoking patients within 30 min of smoking. Cotinine, the primary metabolite of nicotine, has a half-life of approximately 20 h. Serum cotinine levels reflect relatively short-term exposure to cigarette smoke, and they are detectable for up to one week. Therefore, similar to urinary cotinine, serum cotinine is generally used as an objective quantitative indicator of smoking that is more reliable than smoking history [30,31].

Our previous studies using PC9 and HCC827 cell lines showed that an administration of 1–10 μM nicotine induces the lowest level of erlotinib sensitivity, and activates α1nAchR, ERK, AKT, and EGFR. We confirmed the same results in a murine xenograft model [18,19]. In mice, the anticancer effect by erlotinib was reduced by a low-dose, continuous oral administration of nicotine (100 μg/mL) compared with high-dose, rapid intermittent intravenous administration (0.6 mg/kg, five times/week) [19]. This finding indicates that nicotine may reduce the erlotinib anticancer effect in heavy smokers with a longer smoking history.

In the present study, we examined the effects of nicotine on EGFR-TKI under conditions similar to a clinical setting. In both EGFR-TKI-sensitive lung cancer cell lines used in the present study, treatment with serum from smokers resulted in a significant inhibition of the effects of erlotinib, compared with serum from a non-smoker. We also showed that serum from a smoker promotes AKT phosphorylation in PC9 cells when compared to serum from a non-smoker. The results were similar to those of our previous study, in which nicotine was added directly to the culture medium.

Next, we evaluated the effect of serum from smokers on the cell inhibitory effects of other EGFR-TKIs (afatinib and osimertinib) used in varying concentrations, since EGFR-TKIs are sometimes administered at reduced doses clinically. Notably, the results indicated that nicotine inhibited cell growth at all EGFR-TKI concentrations tested. Comparatively, the suppressive effects of serum from a smoker on erlotinib depended on the serum cotinine level. In a previous study, we showed that nicotine promotes the phosphorylation of EGFR/AKT/ERK, and induces resistance to erlotinib in a concentration-dependent manner [19]. In the present study, the observed trend suggested that the blood levels of nicotine affected the development of erlotinib resistance in a concentration-dependent manner.

Lastly, we assessed the resistance to erlotinib therapy by treating cells with serum from a patient treated with erlotinib, along with serum from a smoker. Treatment of PC9 cells with serial dilutions of the serum from the patient treated with erlotinib showed that at all dilutions, the serum from a smoker reduced the cell-inhibitory effects compared with treatment with serum from a non-smoker. An analysis using serum from smokers and a non-smoker indicated that nicotine induces resistance to erlotinib treatment, similar to the results of our previous study.

Smoking cessation and the avoidance of secondhand smoke improve lung cancer treatment in various ways, such as by reducing the risk of secondary cancer, and improving sensitivity to chemotherapy [22]. The present study similarly confirmed the importance of smoking cessation while undergoing EGFR-TKI treatment. It is reported that oxidative stress caused by reactive oxygen species (ROS) such as H_2_O_2_ contained in tobacco smoke causes the abnormal activation of EGFR, and induces resistance to treatment by EGFR-TKI [35,36,37]. Both sera from the smokers used in this study and the cigarette smoke extract (CSE) contained nicotine as well as ROS [37]. Although both components are considered to contribute to the resistance to EGFR-TKI, further investigation with a comparative study on which components contribute the most needs to be performed.

In the future, we must examine how nicotine acts on nAchRs other than the α1 subunit, and investigate components of cigarette smoke other than nicotine.

## 4. Materials and Methods

### 4.1. Cell Culture and Reagents

We purchased PC9 cells from the RIKEN BioResource Center (Ibaraki, Japan), and obtained the HCC827 cells from Kyushu University (Fukuoka, Japan). The cell lines were cultured in RPMI1640 (Gibco, Carlsbad, CA, USA) supplemented with 10% fetal bovine serum (FBS) and 1% penicillin–streptomycin at 37 °C in an atmosphere of 5% CO_2_. Erlotinib was purchased from Cayman Chemical (Ann Arbor, MI, USA). Afatinib and osimertinib were purchased from Selleck Chemicals (Houston, TX, USA).

### 4.2. Serum Collection

Blood was sampled from four smoking patients within 30 min of smoking, one non-smoking patient, and one non-smoking patient with NSCLC who had been treated with erlotinib (150 mg/day) for at least 10 days. Because the inside of the hospital site is a non-smoking site, we collected blood sample from patients within 30 min of them having smoked outside the hospital. The blood samples were centrifuged at 3000 rpm for 20 min, and the serum was collected.

The study involved the secondary use of plasma, and it was approved by the Ethics Committee of the Kyoto Prefectural University of Medicine, Kyoto, Japan (ERB-C-1269, 26/09/2018), and conducted in line with the Declaration of Helsinki.

### 4.3. Measurement of Serum Cotinine Levels

The serum cotinine level, an objective indicator of nicotine exposure [20], was measured in the serum samples from the four smoking patients and one non-smoking patient using a cotinine direct Enzyme-Linked ImmunoSorbent Assay (ELISA) kit (Alere Toxicology, Oxfordshire, UK).

### 4.4. Cell Growth Assay

#### 4.4.1. Evaluation of Inhibitory Effects of Serum from Smokers and EGFR-TKIs

We first seeded PC9 and HCC827 cells at a density of 5000 cells/well in a total volume of 50 μL made up with RPMI1640 supplemented with 10% FBS in a 96-well microplate. The next day, 20 μL serum (either containing or not containing nicotine) from a smoker or the non-smoker was added, followed by the addition of 50 μL erlotinib, afatinib, and osimertinib in RPMI1640 supplemented with 10% FBS to achieve various concentrations (0, 0.1, and 1 μM) of the drugs. Three days later, the numbers of cells were counted by using a cell counting kit (CCK)-F (Dojindo Laboratories, Kumamoto, Japan).

#### 4.4.2. Evaluation of the Inhibitory Effect of Serum from Smokers and Serum from Patients Treated with Erlotinib

First, we added 30 μL serum (either containing or not containing nicotine) from a smoker or the non-smoker to the adjusted PC9 cell suspension (50 μL). The next day, 30 μL serum was added from another patient other than the above, treated with erlotinib, and the resulting suspension was adjusted with RPMI1640 supplemented with 10% FBS to various dilution concentrations (undiluted, diluted at 1:2 and 1:10). Five days later, the numbers of cells were counted using the CCK-F (Dojindo Laboratories).

### 4.5. Western Blotting

Protein aliquots of 9 µg each were resolved by Sodium dodecyl sulfate (SDS) polyacrylamide gel electrophoresis (Bio-Rad, Hercules, CA, USA). After electrophoresis, the protein samples were transferred to polyvinylidene difluoride membranes (Bio-Rad). The membranes were washed three times and incubated with 5% skim milk for 1 h at room temperature, and overnight at 4 °C with the following primary antibodies: p-EGFR, p-Akt (Ser473), t-Akt, β-actin (13E5) (Cell Signaling Technology, Danvers, MA, USA), t-EGFR, p-Erk1/2 (Thr202/tyr204), t-Erk1/2 (R&D systems, Minneapolis, MN, USA). After washing three times, the membranes were incubated for 1 h at room temperature with horseradish peroxidase-conjugated species-specific secondary antibodies (Cell Signaling Technology, Danvers, MA, USA). Immunoreactive bands were visualized using Immobilon Western Chemiluminescent HRP Substrate (Merck Millipore, Darmstadt, Germany).

### 4.6. Statistical Analysis

All data are shown as means ± standard error of the mean (SEM). An analysis between the groups (smokers versus the non-smoker) was conducted using two-way analysis of variance (ANOVA), and *p* < 0.05 was considered to be statistically significant.

## 5. Conclusions

The present study similarly confirmed the importance of smoking cessation while undergoing EGFR-TKI treatment.

## Figures and Tables

**Table 1:** Characteristics and smoking status of four smokers and a non-smoker.

Factor	Non-Smoker		Current Smoker		
Serum No.		1	2	3	4
Gender	Female	Male	Female	Male	Male
Age (years)	57	31	33	35	46
Pack-years	0	1.5	2.5	7.5	18.75
No. of cigarettes/day	0	3	5	10	15
Brinkman index	0	30	50	150	375
Serum cotinine level (ng/mL)	0.6	33.0	65.6	111.6	488.4
